# Annotations

**Published:** 1890-08-30

**Authors:** 


### Annotations.

**Holidays and Health.**

Change of air and scene, of circumstances and of environment generally, is undoubtedly a very good thing, especially for the dwellers in towns, and more particularly for the over-worked dwellers in cities, and for those oppressed by having nothing to do. But very frequently the expected benefit does not result, and for this there are several different reasons. The man who takes his business troubles to the sea-side or into the country can scarcely expect improvement of physical health, while the mind remains too much occupied and disturbed. Neither can the man who is perpetually "running up to town," experience much benefit from sea, mountain, or country air. Again, when the person seeking change is suffering from any ailment considerable attention to the choice of a suitable climate is necessary—an attention which is not always given. It should be recollected that most local guide books—in which the excellences of the local climate are detailed—are written by, or for, interested persons, sometimes for the benefit of railway companies, and at others for the benefit of hotel companies. Again, a locality may be unexceptional with regard to its general character and climate, but there may be numerous positions in such locality where the benefit may be neutralized by various agencies, such as crowded parts, low damp situations, eastern or northern aspect, adjoining massive buildings, closely neighbouring hills, and imperfect ventilation. Again, an invalid requires some sheltered spot for walks or drives, and this is not obtainable at most of our so-called health resorts. It should also be recollected that, particularly at northerly places, cold winds often prevail, and that even in the summer time the English climate is always variable. An erroneous idea prevails that the winds from the sea are not likely to give cold. But a strong sea wind will produce a chill as soon as any other wind, and, although a sea wind loses in harshness, it is often rendered quite as injurious by violence and force. Then, at the sea-side, there may be injudicious bathing. A person may indulge when there are grave reasons why he should not do so; or he may remain in the water so long that he is injuriously affected. Then the capacity of hotels and lodging houses is often so stretched, that they are made to contain many more inmates than can be satisfactorily accommodated. There is not only overcrowding, but the means provided for the disposition of waste is over-taxed, perhaps leading to soil saturation and water pollution. Healthy homes are left behind, and often the result is no benefit experienced from the change. There is also another evil. Houses are vacated or even closed for weeks or months, and no provision is made for the sanitation thereof. The family may therefore return to stagnant water, to escapes of sewer gas, to rooms which have not been ventilated during their absence, and to beds which have absorbed moisture while unoccupied. All these things demand thought and intelligence.

**A New Home for Epileptics.**

No cases with which medical men have to deal are more grievous than those of epileptics, and no class of sufferers is so poorly provided for. In the whole of Great Britain there are probably not half a dozen hospitals or homes set apart for the treatment of those unhappy persons. Burdett's *Hospital Annual* mentions only three such homes. And yet no cases are more capable of decided and permanent improvement under proper treatment. Those who know about the subject are exceedingly glad to see that a new home has been commenced at Maghull, near Liverpool. It is as yet the day of small things with the new institution; but the first annual report recently published, is highly satisfactory. To Dr. William Alexander belongs the honour of having started the institution, and to "a friend of Dr. Alexander" appertains the equal, if not greater, honour of having provided a sum of £1,500 in order to give the experiment a fair trial. The institution was opened on December 28th, 1888, with a single patient. Within a year thirty additional patients had been admitted; and there were twenty-two in the hospital at the date of the issue of the report. Many people, who think that charitable institutions are overdone in this country, will be surprised to learn that the donations and subscriptions which came in during the first year amounted to over £2,300, whilst the patients themselves contributed about £460, making together an annual income of nearly £2,800. This result of a single year's work is both surprising and gratifying. The number of patients received proves beyond a doubt the urgent necessity there is for a large increase of resources for the care of epileptics, and the liberal donations given to a comparatively unknown and untried institution shows that the public is thoroughly aware of the need, and is well disposed to try to meet it. It is much to be desired that the example of Maghull will stimulate medical men and wealthy persons all over the country, and that we may soon have as many homes for epileptics as are required.

**The Dangers of Tuberculous Milk.**

It is a well established fact that cattle are frequently affected with tuberculosis, and die of the disease. Persons not accustomed to the ways of doctors are apt to think that they may be mistaken when they speak of tuberculous cattle, and the dangers which result to men, women, and children from such cattle. But there is no more difficulty in diagnosing consumption in a cow that in a human being. It is quite easy to see that a particular animal is wasting away. It is just as easy to observe that it is short of breath and has a bad cough. Those symptoms are as patent to the farmer as to the doctor. When in addition the medical man uses his stethoscope, his percussion, his thermometer, and all the other methods of a specialist, the nature of the disease is established beyond a doubt. But if any critic still remains sceptical the dead-house offers testimony which cannot for a moment be called in question. Here, for example, is the clinical record of the case of a tuberculous cow: On June 15th, 1878, an experienced veterinary surgeon was called to see a red and white cow. The animal had a cough and was short of breath; its temperature was 104 deg. Percussion and auscultation showed that one part of the right lung was too solid, and that another part had a cavity in it. The surgeon expressed his opinion that the cow was tuberculous, and advised that she should be destroyed. She was not destroyed; on the contrary, she was kept and milked, and the family used the milk. Six months later the same veterinary surgeon was called to visit the cow, and found her with a higher temperature, a quicker pulse, and more rapid breathing. Every sign of tuberculosis was more marked; and again the destruction of the animal was advised. Three months later a further visit showed rapidly advancing tuberculosis with much emaciation. The family still continued to use the milk, although on the occasion of each visit they had been advised not to do so. At the end of May, 1879, that is a little less than a year from the first visit, the surgeon was called in a great hurry. The cow was evidently dying, and did die in three hours. There was now an opportunity of proving or disproving the tuberculosis theory by means of a post-mortem examination. The examination showed that both lungs were infiltrated with tubercle; and not only so, but that the kidneys, intestines, udder, and other parts, including the flesh, were in a tuberculous condition. Now the object of giving this history at some length is to relate what followed, and to found an important practical conclusion thereupon. Within three months of the death of the cow the farmer's baby was taken sick and died. A post-mortem examination was made, which proved that the cause of death was tubercle of the brain and lung. More than a year after that a second child died, also from tubercle of the lung. Those children had not eaten any part of the flesh of the cow, of course, but they had drunk the milk. The moral certainty was that the milk of the tuberculous cow had been the cause of the rapid consumption in the children; for both parents and grand parents were still alive at the time of their death, and no previous case of consumption had ever been heard of in the family. There are three practical lessons to be learnt from this history. The first is that cattle which are reasonably suspected of being tuberculous should be destroyed at the cost of the state; the second that milk from sickly animals should never be drunk at all, much less when those animals are suspected of tuberculosis; and the third, that the flesh of such animals should never on any account be eaten. Some competent observers believe that tuberculosis originated with cattle, and that it would be entirely stamped out by carefully isolating all the human beings that are consumptive, and killing, and destroying the flesh of all the cattle that show any signs of the same affection. That, we consider, is going too far, but the whole subject is well worthy of the serious consideration of both medical scientists and the public at large.

